# Validation and application of a prognostic model for patients with advanced pancreatic cancer receiving palliative chemotherapy

**DOI:** 10.1002/cam4.2483

**Published:** 2019-08-06

**Authors:** Pei‐Wei Huang, Ching‐Fu Chang, Chia‐Yen Hung, Shun‐Wen Hsueh, Pei‐Hung Chang, Kun‐Yun Yeh, Jen‐Shi Chen, Yen‐Yang Chen, Chang‐Hsien Lu, Yu‐Shin Hung, Wen‐Chi Chou

**Affiliations:** ^1^ Division of Hematology‐Oncology Department of Internal Medicine Chang Gung Memorial Hospital at Linkou and Chang Gung University College of Medicine Taoyuan Taiwan; ^2^ Division of Hematology‐Oncology Department of Internal Medicine Mackay Memorial Hospital Taipei Taiwan; ^3^ Division of Hematology‐Oncology Department of Internal Medicine Chang Gung Memorial Hospital at Keelung Keelung Taiwan; ^4^ Division of Hematology‐Oncology Department of Internal Medicine Chang Gung Memorial Hospital at Kaohsiung Kaohsiung Taiwan; ^5^ Division of Hematology‐Oncology Department of Internal Medicine Chang Gung Memorial Hospital at Chiayi Chiayi Taiwan

**Keywords:** palliative chemotherapy, pancreatic cancer, prognostic model

## Abstract

**Background:**

We previously developed a robust prognostic model (GS model) to predict the survival outcome of patients with advanced pancreatic cancer (APC) receiving palliative chemotherapy with gemcitabine plus S‐1 (GS). This study aimed to validate the application of the GS model in APC patients receiving chemotherapy other than the GS regimen.

**Patients and methods:**

We retrospectively analyzed 727 APC patients who received first‐line palliative chemotherapy other than the GS regimen between 2010 and 2016 at four institutions in Taiwan. The patients were categorized into three prognostic groups based on the GS model for comparisons of survival outcome, best tumor response, and in‐group survival differences with monotherapy or combination therapy.

**Results:**

The median survival times for the good, intermediate, and poor prognostic groups were 13.4, 8.4, and 4.6 months, respectively. The hazard ratios for the comparisons of intermediate and poor to good prognostic groups were 1.51 (95% confidence interval [CI]), 1.22‐1.88, *P* < .001) and 2.84 (95% CI, 2.34‐3.45, *P* < .001). The best tumor responses with either partial response or stable disease were 57.5%, 40.4%, and 17.2% of patients in the good, intermediate, and poor prognostic groups (*P* < .001), respectively. For patients in the good prognostic group, first‐line chemotherapy with monotherapy and combination therapy had similar median survival times (13.8 vs 12.9 months, *P* = .26), while combination therapy showed a better median survival time than monotherapy in patients in the intermediate and poor prognostic groups (8.5 vs 8.0 months, *P* = .038 and 5.7 vs 3.7 months, *P* = .001, respectively).

**Conclusion:**

The results of our study supported the application of the GS model as a general prognostic tool for patients with pancreatic cancer receiving first‐line palliative chemotherapy with gemcitabine‐based regimens.

## Graphical abstract

This study provided validation of the previously developed gemcitabine + S‐1 model in predicting the survival outcome of advanced pancreatic cancer patients receiving first‐line palliative chemotherapy.

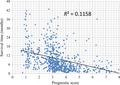

## 1 INTRODUCTION

Pancreatic cancer is one of the most lethal cancers worldwide; as more than 80% of patients present with unresectable disease at diagnosis,^1^, ^2^ palliative chemotherapy is the standard of care.^3^ Gemcitabine has been the backbone of chemotherapy for advanced pancreatic cancer (APC) for several decades with clinical benefits of prolonging survival time and ameliorating patient quality of life 4; however, gemcitabine monotherapy has demonstrated a tumor response rate of 6%‐11% and a median survival time of 5.6‐8.8 months in APC.^4^, ^5^, ^6^ Recent clinical studies using combination regimens including 5‐flourouracil (5‐FU) plus folinic acid, irinotecan, and oxaliplatin (FOLFIRINOX) or gemcitabine plus nab‐paclitaxel have shown improved survival outcomes compared to those of gemcitabine monotherapy.^5^, ^6^ Even though patients received high‐intensity treatment, the median survival times were around 11.1 months in patients treated with FOLFIRINOX and 8.5 months in patients treated with gemcitabine plus nab‐paclitaxel.^5^, ^6^ Unfortunately, these high‐intensity treatments are inevitably associated with higher treatment‐related toxicity.^7^, ^8^ For example, grade 3 or 4 neutropenia occurred in 45% of patients treated with FOLFIRINOX and 38% of patients treated with gemcitabine plus nab‐paclitaxel.^5^, ^6^ Due to the marginal survival benefits and increased toxicity profiles, early meta‐analysis studies suggested that combination therapies should be reserved for APC patients with good performance status.^7^, ^8^, ^9^, ^10^

While palliative chemotherapy has shown prolonged survival,^4^, ^5^, ^6^ there is an essential need to identify APC patients who might benefit the most and the least from such treatment since chemotherapy is often accompanied by extreme toxicity.^4^, ^5^, ^6^, ^7^, ^8^, ^9^, ^10^ We previously developed a robust prognostic model (the GS model) 11 to predict survival outcomes of APC patients who received first‐line palliative chemotherapy with gemcitabine plus S‐1 (GS regimen).^12^ Four independent variables—performance status, tumor stage, albumin, and neutrophil‐to‐lymphocyte ratio (NLR)—were selected to construct the model, which categorized patients into different prognostic groups for survival prediction. The clinical variables of the GS model are easily accessed upon antitumor therapy; therefore, we believed that this model might be applicable to patients with APC receiving all chemotherapeutic regimens. The GS prognostic model was originally designed to predict survival outcome in APC patients who received first‐line palliative chemotherapy with GS regimen. The primary outcome of this study aimed to validate the performance and validate the application of the GS model to APC patients receiving first‐line palliative chemotherapy regimens other than GS.

## 2 MATERIALS AND METHODS

### 2.1 Patient selection

We constructed a retrospective medical chart review to select consecutive patients who underwent first‐line palliative chemotherapy for unresectable or metastatic pancreatic cancer (APC) between 2010 and 2016 at four institutions in Taiwan. Patients who had recurrent tumor after receiving adjuvant chemotherapy, experienced a concurrent active malignancy, received GS regimen or concomitant radiotherapy during first‐line palliative chemotherapy were excluded. A total of 727 patients who received palliative chemotherapy other than the GS regimen as the first‐line treatment were included in this study. The choice of chemotherapy regimens was determined by the primary care physician on the basis of the patients and physicians' preference. S‐1 is an oral 5‐FU derivative and has been widely used in Asian countries for treating pancreatic cancer according to Gemcitabine and S‐1 Trial (GEST) study.^12^ S‐1 monotherapy was provided at a dose of 80‐120 mg daily on day 1 to day 14 every 21 days or on day 1 to day 28 every 42 days.^12^ This study was approved by the institutional review boards and was conducted in compliance with the principles of the Declaration of Helsinki (1996). The requirement of informed consent from participants was waived by the Institutional Review Board because no protected health information was included in this retrospective study.

### 2.2 Data collection and follow‐up

Patient demographic data were recorded by primary care physicians using a prospectively formulated electronic data form described in detail elsewhere.^8^ All biochemistry laboratory data were obtained within 7 days prior to chemotherapy initiation. Monotherapy and combination therapy were respectively defined as the administration of one and more than one kind of chemotherapeutic agent. Tumor response was evaluated using computed tomography (CT) scan according to the Response Evaluation Criteria in Solid Tumors (RECIST) 1.1.^13^ Image studies were performed and interpreted by an institutional radiologist at baseline and were repeated every 12 weeks (the exact interval was at the physician's discretion) to evaluate tumor response. Overall survival (OS) was calculated from the time of chemotherapy initiation until the date of death from any cause. All included patients were followed‐up until death or 31 December 2017. All dates of death were obtained from either the Institutional Cancer Registry or the National Registry of Death database in Taiwan.

### 2.3 Statistical analysis

Basic demographic data were summarized as n (%) for categorical variables and as medians with 95% confidence intervals (CIs) for continuous variables. Four predefined variables—tumor stage, performance status, albumin, and NLR—selected from the GS model,^11^ were evaluated using univariate Cox regression to assess the impact of each variable on patient OS. All four variables were further analyzed using a multivariate Cox proportional hazards model with backward selection.

The prognostic score was calculated for each patient using the GS model, as shown below:

**Prognostic score = 3 + (0 for ECOG PS 0 or 1; 0.359 for ECOG PS 2; 2.709 for ECOG PS 3) + (0 for stage III; 1.636 for stage IV) + 0.186 × NLR (100%)−0.547 × albumin (gm/dL)**

The patients were categorized into good (prognostic score <2.7), intermediate (prognostic score 2.7‐3.3), and poor prognostic groups (prognostic score >3.3) according to the GS model. Survival time was analyzed using the Kaplan‐Meier method. Log‐rank tests were used to determine significant differences among the survival curves. The C‐index was calculated to assess the performance of the GS model. The c‐index is a generalization of the area under the receiver operating characteristic curve, which measures the discrimination of model. The value of c‐index ranges from 0.5 (no discrimination) to 1.0 (perfect discrimination). All statistical assessments with *P* < .05 were considered significant.

## 3 RESULTS

### 3.1 Patient characteristic

Comparisons of the distributions of patient characteristics in the study cohort (non‐GS cohort) and the cohort used in the development of the GS model (GS cohort) are shown in Table 1. In the non‐GS cohort, the median age was 63 years (range, 23‐89) and 431 patients (59.3%) were men. In this cohort, 565 patients (77.7%) had metastatic disease with the liver as the most common metastatic site (52.4%), followed by the peritoneum (28.6%) and distant lymph nodes (18.3%). The most common chemotherapy regimen used for first‐line treatment was gemcitabine‐based combination therapy (50.6%), followed by gemcitabine alone (36.3%), 5‐FU or S‐1 alone (9.5%), and combination therapy with miscellaneous agents (3.6%). Less than 1% of non‐GS cohort patients had been treated with gemcitabine + Nab‐paclitaxel or FOLFIRINOX regimen because of a lack of insurance support. Compared to the demographic data of patients in the GS cohort, patients in the non‐GS cohort were characterized by higher proportions of nonsmoking patients and those with primary tumors located at the pancreatic head.

**Table 1:** Patient characteristics

Characteristic	Non‐GS cohort (n = 727), N (%)	GS cohort (n = 111), N (%)	*P*‐value
Median age, y (range)	63 (23‐89)	62 (32‐82)	0.56^a^
Male sex	431 (59.3)	66 (59.5)	0.99^b^
Median BMI, kg/m^2^ (range)	22.7 (13‐36.2)	22.5 (15.6‐32.5)	0.76^a^
ECOG performance status			
0 or 1	508 (69.9)	89 (80.2)	0.08^b^
2	187 (25.7)	19 (17.1)	
3	32 (4.4)	3 (2.7)	
Charlson comorbidity index			
0	196 (27.0)	31 (27.9)	0.63^b^
1	253 (34.8)	39 (35.1)	
2	172 (23.7)	21 (18.9)	
>2	106 (14.6)	20 (18.0)	
Smoking			
Never	47 (65.5)	56 (50.5)	**0.003** b
Ever or active	251 (34.5)	55 (49.5)	
Primary tumor site			
Head	311 (42.8)	32 (28.8)	**0.017** b
Body	124 (17.1)	24 (21.6)	
Tail	139 (19.1)	32 (28.8)	
Overlapping	153 (21.0)	23 (20.7)	
Presence of jaundice			
No	482 (66.3)	84 (75.7)	0.06^b^
Yes	245 (33.7)	27 (24.3)	
T‐classification			
1	11 (1.5)	2 (1.8)	0.34^b^
2	68 (9.4)	14 (12.6)	
3	159 (21.9)	30 (27.0)	
4	489 (67.3)	65 (58.6)	
N‐classification			
0	179 (24.6)	19 (17.1)	0.10^b^
1	548 (75.4)	92 (82.9)	
M‐classification			
0	162 (22.3)	22 (19.8)	0.63^b^
1	565 (77.7)	89 (80.2)	
AJCC tumor stage			
III	162 (22.3)	22 (19.8)	0.63^b^
IV	565 (77.7)	89 (80.2)	
Median albumin, gm/dL (range)	3.80 (1.3‐4.9)	4.0 (2.7‐4.9)	0.37
Median NLR (range)	3.7 (0.7‐43)	3.3 (0.8‐17)	0.10
Site of metastases			
Liver	381 (52.4)	57 (51.4)	0.84^b^
Peritoneum	208 (28.6)	31 (27.9)	0.99^b^
Distant lymph nodes	133 (18.3)	17 (15.3)	0.51^b^
Lung	85 (11.7)	13 (11.7)	0.99^b^
Others	47 (6.5)	4 (3.6)	0.29^b^
First‐line chemotherapy regimen			
GS regimen	…	111 (100)	
Gemcitabine‐based			
Combination	365 (50.2)	…	
Gemcitabine + cisplatin	245 (33.7)		
Gemcitabine + 5‐FU	63 (8.7)		
Gemcitabine + oxaliplatin	51 (7.0)		
Gemcitabine + erlotinib	6 (8.0)		
Gemcitabine + Nab‐paclitaxel	3 (0.4)	…	
FOLFIRINOX regimen	4 (0.6)	…	
Combination therapy with miscellanea agents	22 (3.0)	…	
5‐FU + cisplatin	14 (1.9)		
5‐FU + oxaliplatin	5 (0.7)		
5‐FU + irinotecan	3 (0.4)		
Gemcitabine alone	264 (36.3)	…	
5‐FU or S‐1 alone	69 (9.5)	…	

Abbreviations: 5‐FU, 5‐fluouracil; AJCC, American Joint Committee on Cancer; BMI, body mass index, ECOG, Eastern Cooperative Oncology Group; FOLFIRINOX, 5‐fluouracin plus folinic acid, irinotecan, and oxaliplatin; GS, gemcitabine plus S‐1; NLR, neutrophil‐to‐lymphocyte ratio.

a Mann‐Whitney U test.

b Chi‐square test.

Bold values indicate *P* < 0.05.

### 3.2 Survival outcome and performance of the GS model

The median OS time was 7.4 months (95% CI, 7.0‐7.9) and 57 (7.8%) patients remained alive at the end of the study. Table 2 shows the results of univariate and multivariate analyses of the variables within the GS model for OS. All four variables, (ECOG performance, tumor stage, albumin level, and NLR) were significant prognostic factors for OS in both univariate and multivariate analysis.

**Table 2:** Univariate and multivariate analysis of variables in the GS model for overall survival

Variable	Category	Univariate analysis		Multivariate analysis	
		HR (95% CI)	*P*‐value^a^	Adjusted HR (95% CI)	*P*‐value^a^
ECOG PS	0 or 1	1		1	
	2	2.49 (2.09‐2.98)	<.001	2.15 (1.78‐2.60)	<.001
	3	4.41 (2.99‐6.50)	.011	4.20 (2.79‐6.35)	<.001
Tumor stage	III	1		1	
	IV	1.84 (1.52‐2.22)	<.001	1.81 (1.49‐2.21)	<.001
Albumin	per g/dL	0.60 (0.53‐0.68)	<.001	0.82 (0.72‐0.94)	.004
NLR	per ratio	1.04 (1.02‐1.05)	<.001	1.02 (1.01‐1.04)	.009

Abbreviations: HR, hazard ratio; CI, confidence interval; ECOG PS, Eastern Cooperative Oncology Group performance scale; NLR, neutrophil‐to‐lymphocyte ratio.

a Cox model analysis.

**Figure 1:**
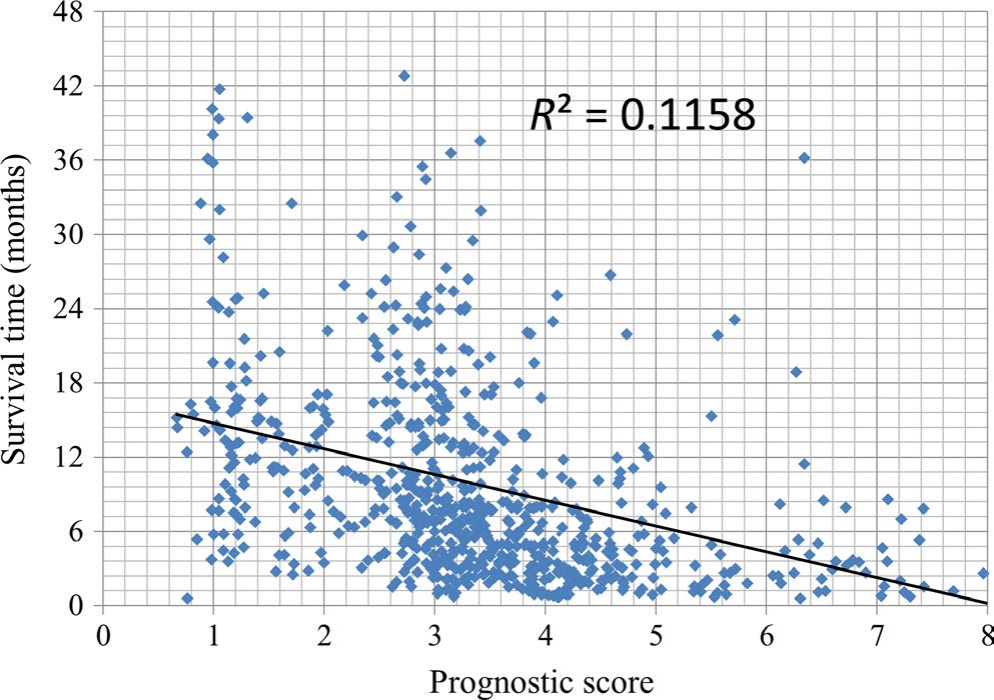
Scatter plot showing a linear correlation between prognostic score and survival time

Figure 1 shows the linear correlation between the GS model scores and survival times (R^2^ = 0.116). Patients with a lower prognostic score had a longer survival time. The C‐index of the GS model applied to the non‐GS cohort was 0.73 (95% CI, 0.70‐0.77).

**Figure 2:**
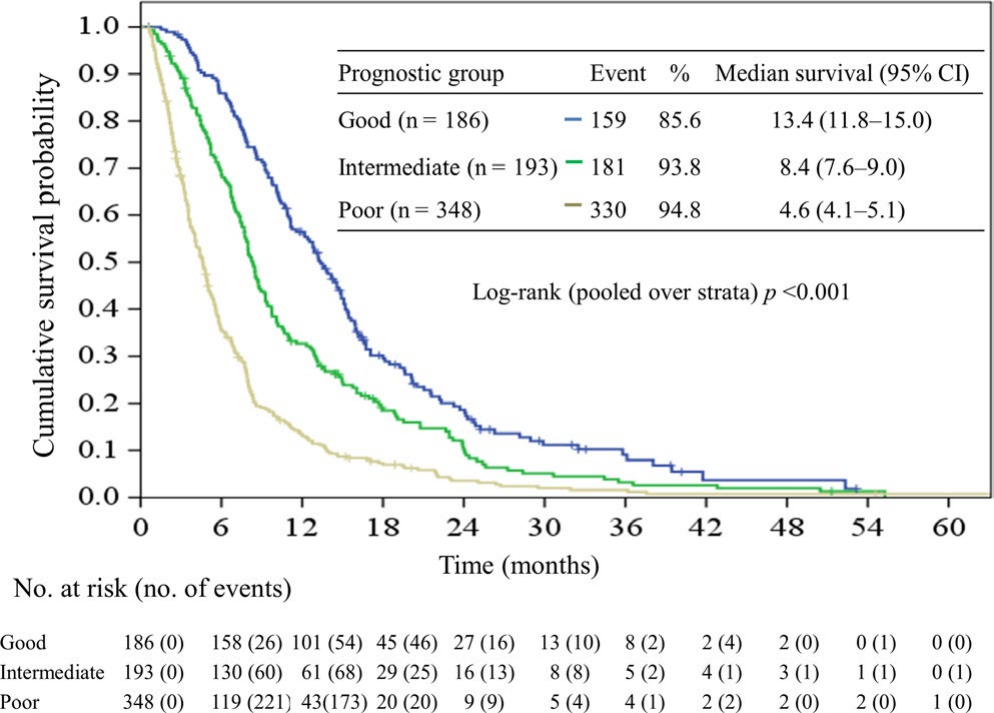
Kaplan‐Meier plot of overall survival in patients stratified by prognostic group

Based on the GS model, 32.4%, 29.7%, and 37.8% of the patients were assigned to the good, intermediate, and poor prognostic groups, respectively. The median survival times for these three groups were 13.4 (95% CI, 11.8‐15.0), 8.4 (95% CI, 7.7‐9.0), and 4.6 (95% CI, 4.1‐5.1) months, respectively. Kaplan‐Meier survival curves of patients from different prognostic groups are shown in Figure 2. The hazard ratios for the comparison of the intermediate and poor prognostic groups to the good prognostic group were 1.51 (95% CI, 1.22‐1.88, *P* < .001), and 2.84 (95% CI, 2.34‐3.45, *P* < .001), respectively (Table 3). The performance of the GS model among patients receiving different chemotherapy regimens is shown in Table 3. Overall, the OS was distinctively longer in the good prognostic group than that in the poor prognostic group across different chemotherapeutic regimens (Figure S1).

**Table 3:** Subgroup analysis for survival based on GS prognostic group by different treatment regimen

Treatment regimen	Prognostic group	N (%)	Median OS, months (95% CI)	HR (95% CI)	*P*‐value^a^
Overall (n = 727)	Good	186 (32.4)	13.4 (11.8‐15.0)	1 (reference)	
	Intermediate	193 (29.7)	8.4 (7.7‐9.0)	1.51 (1.22‐1.88)	<.001
	Poor	348 (37.8)	4.6 (4.1‐5.1)	2.84 (2.34‐3.45)	<.001
Gemcitabine‐based or miscellaneous agents combination (n = 394)	Good	106 (28.8)	12.8 (10.4‐15.2)	1 (reference)	
	Intermediate	119 (32.3)	8.7 (7.7‐9.7)	1.39 (1.06‐1.83)	.019
	Poor	143 (38.9)	5.8 (5.0‐6.6)	2.42 (1.85‐3.16)	<.001
Gemcitabine alone (n = 264)	Good	53 (20.1)	13.6 (11.7‐15.5)	1 (reference)	
	Intermediate	52 (19.6)	7.7 (6.3‐9.2)	1.70 (1.13‐2.54)	.011
	Poor	159 (60.2)	3.7 (3.1‐4.2)	3.00 (2.13‐4.24)	<.001
5‐FU or S‐1 alone (n = 69)	Good	20 (29.0)	15.2 (9.0‐21.4)	1 (reference)	
	Intermediate	10 (14.5)	8.8 (2.8‐14.3)	1.74 (0.73‐4.10)	.21
	Poor	39 (56.5)	3.7 (2.7‐4.7)	2.99 (1.63‐5.50)	<.001

Abbreviations: 5‐FU, 5‐fluouracil; CI, confidence interval; HR, hazard ratio; OS, overall survival.

a Cox model analysis.

**Figure 3:**
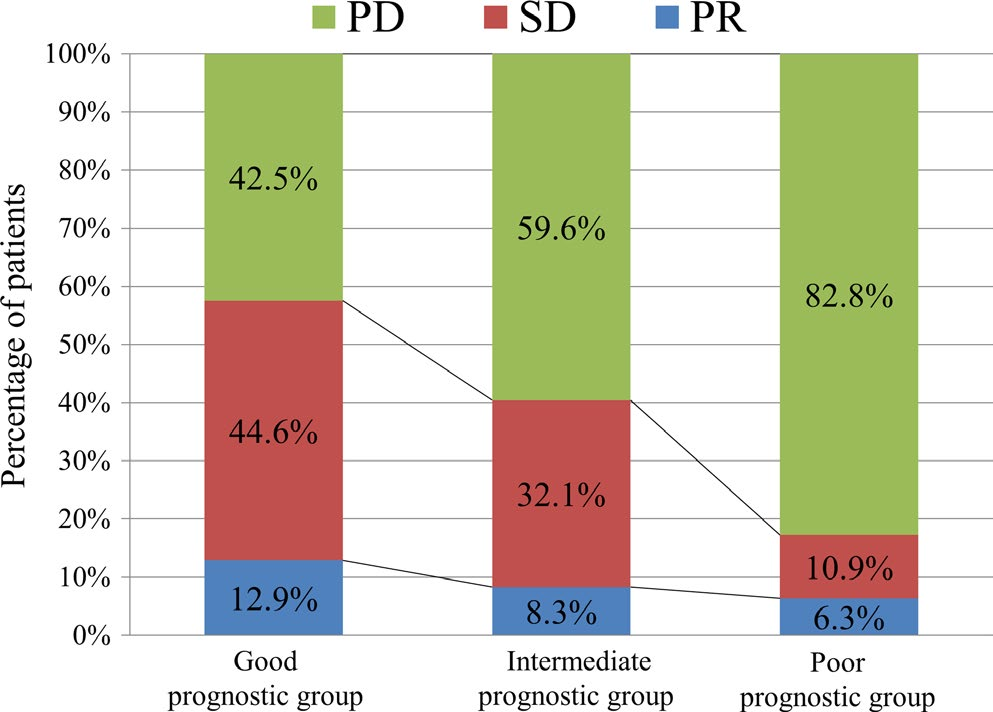
Best tumor response to chemotherapy, stratified according to the GS model. PD, progressive disease; PR, partial response; SD, stable disease

Of the non‐GS cohort, first‐line chemotherapy showed the best treatment response of partial response (PR) in 62 (8.5%) patients, stable disease (SD) in 183 (25.2%) patients, and progressive disease (PD) in the remaining 482 (66.3%) patients. The best treatment responses of PR, SD, and PD occurred in 12.9%, 44.6%, and 42.5% of patients in the good prognostic group; 8.3%, 32.1%, and 59.6% of patients in the intermediate prognostic group; and 6.3%, 10.9%, and 82.8% of patients in the poor prognostic group, respectively (chi‐square test *P* < .001, Figure 3).

### 3.3 Impact of monotherapy or combination therapy among prognostic groups

**Figure 4:**
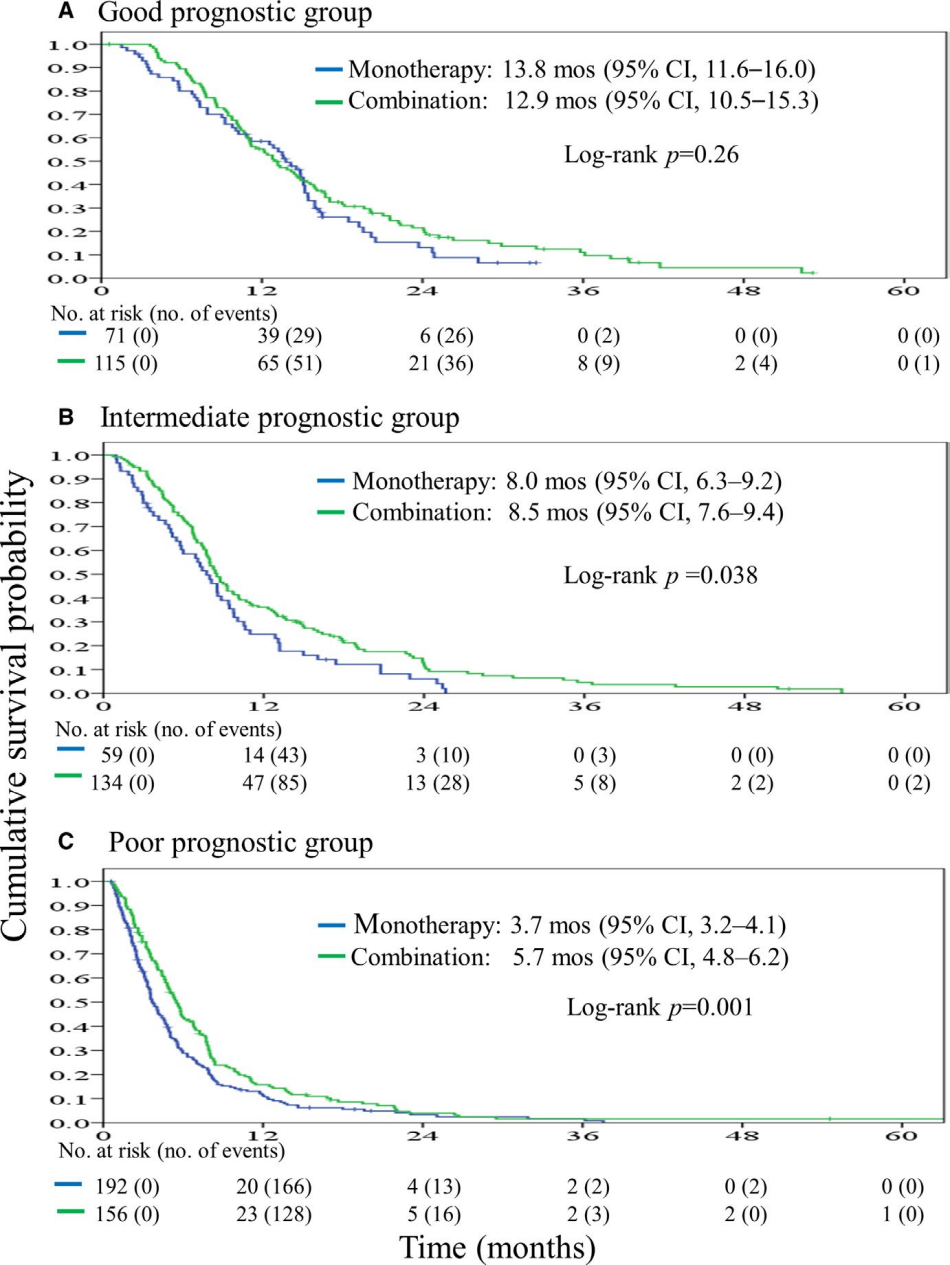
In‐group survival differences of patients with advanced pancreatic cancer administered monotherapy or combination therapy. A, Patients in the good prognosis group. B, Patients in the intermediate prognosis group. C, Patients in the poor prognosis group

To evaluate the impact of chemotherapy regimen on survival outcome among the different prognostic groups, all patients were categorized as having received either monotherapy or combination therapy as the first‐line treatment for pancreatic cancer. In the good prognostic group, the median OS were 13.8 (95% CI, 11.6‐16.0 months) and 12.9 (95% CI, 10.5‐15.3 months, *P* = .26) months for patients treated with monotherapy and combination therapy, respectively (Figure 4A). In the intermediate prognostic group, the median OS were 8.0 (95% CI, 6.4‐9.5 months) and 8.5 (95% CI, 7.5‐9.5 months, *P* = .038) months for patients with monotherapy and combination therapy, respectively (Figure 4B). In the poor prognostic group, the median OS were 3.7 (95% CI, 3.2‐4.1 months) and 5.7 (95% CI, 5.0‐6.4 months, *P* = .001) months for patients treated with monotherapy and combination therapy, respectively (Figure 4C).

## 4 DISCUSSION

This study provided validation of the previously developed GS model in predicting the survival outcome of APC patients receiving first‐line palliative chemotherapy. In addition to survival outcome prediction, our data demonstrated the outstanding predictive ability of treatment response with the GS model. Our current study extended the clinical application of the GS model for survival prediction regardless of the chemotherapy regimen used for the treatment of patients with APC.

In line with the GS model,^11^ the current study helped identify performance status, tumor stage, albumin, and NLR as independent prognostic factors that predict survival outcome. Several prognostic models have been developed for survival prediction among APC patients receiving first‐line palliative chemotherapy.^14^, ^15^, ^16^, ^17^, ^18^, ^19^, ^20^, ^21^, ^22^, ^23^, ^24^, ^25^ Apart from patient factors (age, sex, performance status, weight loss), laboratory variables (albumin, white cell count, NLR, tumor markers) and tumor characteristics (stage, tumor size, metastatic organ, ascites) have all been reported to be independent prognosticators in different prognostic models.^14^, ^15^, ^16^, ^17^, ^18^, ^19^, ^20^, ^21^, ^22^, ^23^, ^24^, ^25^ Although no consistent prognosticators were reported among different reports, all models showed that in addition to tumor factors, patient features also were relevant to prognosis in PC patients. Consequently, the GS model was constructed by the combination of tumor‐ and patient‐related factors, which may be more effective at predicting survival outcome than either tumor or patient factors alone.

Table 4 summarizes and compares 13 published prognostic models for the prediction of the survival outcome of PC patients receiving palliative chemotherapy from a literature review.^14^, ^15^, ^16^, ^17^, ^18^, ^19^, ^20^, ^21^, ^22^, ^23^, ^24^, ^25^ Only one of the models was designed prospectively 25; the others were retrospective studies. Of the 13 prognostic models, some were used for the prediction of survival outcome specifically for gemcitabine‐based treatment,^11^, ^14^, ^16^, ^19^, ^22^, ^24^, ^25^ while the other models consisted a broad spectrum of chemotherapeutic regimens.^15^, ^17^, ^18^, ^20^, ^21^, ^23^ The performance reflected by C‐index ranged from 0.66 to 0.80 among these prognostic models. Only five models were validated by application to a different cohort.^14^, ^17^, ^23^, ^25^ Because the GS model is easily applied and had good performance in term of C‐index value in the original and the current cohort, we believe this model to be widely applicable by clinicians to predict survival outcome in APC patients receiving first‐line palliative chemotherapy.

**Table 4:** Comparisons of prognostic models of patients with advanced pancreatic cancer receiving first‐line palliative chemotherapy

Study	Year	Country	Number of patients	Characteristics	Chemotherapy regimen	Validation	Prognostic factors	C‐index
Yi, et al 15	2011	Korea	298	Retrospective	Gemcitabine‐based	No	Liver metastasis, ascites or carcinomatosis, CRP, albumin	NR
Inal, et al 22	2012	Turkey	343	Retrospective	Gemcitabine or Gemcitabine plus cisplatin	No	Stage, weight loss, CEA	NR
Hamada, et al 24	2014	Japan	531	Retrospective	Gemcitabine‐based	No	Age, sex, PS, tumor size, regional lymph node metastasis, distant metastasis	0.686
Xue, et al 15	2015	China	118	Retrospective	Gemcitabine‐ or S‐1‐based	No	PS, CA19‐9, CRP	NR
Kurihara, et al 20	2015	Japan	182	Retrospective	Gemcitabine plus S‐1	No	PS, Stage, ANC	NR
Kou, et al 21	2016	Japan	306	Retrospective	Gemcitabine monotherapy, S‐1 monotherapy, gemcitabine plus S‐1, and gemcitabine plus erlotinib	No	PS, distant metastatic disease, initially unresectable disease, CEA, CA 19‐9, Neutrophils and lymphocytes	0.658
Xue, et al 17	2017	China Japan	153 (training set) 252 (validation set)	Retrospective	All	Yes	Lymphocytes and monocytes	NR
Deng, et al 25	2017	China	1017 (training set) 509 (validation set	Prospective, randomly assign	Gemcitabine‐based	Yes	Age, tumor stage, tumor size, ALT, albumin, CA 19‐9, hepatitis B virus infection status	0.720 (training set) 0.696 (validation set)
Ventriglia, et al 19	2018	Italy	70	Retrospective	Nab‐paclitaxel plus gemcitabine	No	PS, liver metastasis, NLR	NR
Hang, et al 23	2018	China	133 (training cohort) 273 (validation cohort)	Retrospective from three clinical trials	NR	Yes	PS, liver metastasis, CA 19‐9, ANC, albumin	0.699 (training cohort) 0.658 (validation cohort)
Wang, et al 18	2018	China	94	Retrospective	Gemcitabine or 5‐FU‐based,	No	LDH, CA19‐9, CRP, albumin	NR
Zhang, et al 14	2019	China	197 (training cohort) 222 (validation cohort)	Retrospective	NR	Yes	Platelet, neutrophil, lymphocyte counts	NR
Chang, et al 11	2019	Taiwan	111	Retrospective	Gemcitabine + S‐1	Yes	PS, stage III or IV, neutrophil, lymphocyte, albumin	0.80
Current study		Taiwan	727	Retrospective	All regimen	…	…	0.73

Abbreviations: ALT, alanine aminotransferaseANC, absolute neutrophil count; CA19‐9, carbohydrate Antigen 19‐9; CEA, carcinoembryonic antigen; CRP, C‐reactive protein; NLR, neutrophil‐to‐lymphocyte ratio; NR, not recorded; PS, performance status.

Due to the increased toxicity profiles in combination chemotherapy, meta‐analyses have indicated that such treatments might provide survival benefit only in APC patients with a good performance status and might be harmful in these with a poor performance status.^7^, ^8^, ^9^, ^10^ The gemcitabine and S‐1 Trial (GEST) study was a phase III trial conducted to compare the clinical efficacy of S‐1 monotherapy, gemcitabine monotherapy, and S‐1 and gemcitabine combined therapy as first‐line chemotherapy for APC.^12^ The study results revealed nonsignificant differences in survival between the three treatment groups, especially for patients with a performance status of 0. However, in patients with a performance status of 1, GS combination therapy provided a longer survival time than that of monotherapy (median OS 9.6 months for GS, 6.2 months for gemcitabine, and 6.3 months for S‐1).^26^ In line with those of the GEST,^12^ our results showed similar survival outcomes for patients in the good prognostic group regardless of treatment with monotherapy or combination therapy; in contrast, patients in the intermediate or poor prognostic groups receiving monotherapy had distinctively worse survival than those receiving combination therapies. Due to conflicting results regarding the impact of combination therapy or monotherapy on survival in patients with poor performance status, a well‐designed prospective study may help to elucidate the effect of these therapies on survival outcome in APC patients.

The R‐square value for the GS model in our patient cohort was 0.11, suggesting an unsatisfied linear correlation of the GS prognostic score and OS. However, a significant in‐groups survival difference supported that the prognostic groups stratification according to the GS model was accurate to provide survival discrimination. The results of the current study supported the use of the GS model for guidance in treatment planning for APC patients. With the intent to prolong survival outcome, patients in the intermediate or poor prognostic groups in the GS model should be encouraged to receive combination therapy rather than monotherapy. Nonetheless, patients should also be informed that the gain of survival benefit is potentially accompanied by additional toxicity from the combination therapies.

The strength of this study rests in its having the largest cohort among currently published prognostic studies used for the validation of a robust prognostic model. Despite some differences in clinical parameters between the non‐GS and GS cohorts, the results of the current study supported the generalization of the GS model as an accurate prognostic tool in patients with APC. Because all the parameters of the GS model are easily accessible, objective, and available upon initiation of systemic chemotherapy, we believe that it could be widely applied as a simple prognostic tool for APC patients receiving all types of first‐line chemotherapy regimens. The current study not only includes large patient samples, most importantly, but also validates the model performance by allocated patients according to different chemotherapy regimens. However, the current study had several limitations. First, our study is susceptible to selection bias due to its retrospective approach. Second, a long initial “plateau” for patients in the good prognostic group in the Kaplan‐Meier plots might raise the concerns of a survival bias. Malnutrition and infection are the main leading causes of death in patients with pancreatic cancer.^3^ Patients in good prognostic group possess better general condition in terms of good performance and higher albumin level against these threats and may tolerate anticancer treatment better. This could be the cause of a long initial “plateau” for patients in the good prognostic group in the Kaplan‐Meier plots. A prospective study is needed to eliminate questions about the selection and survival bias in this study. Third, since the choice of chemotherapy regimen and dosing is individualized by physician and patient preferences, personalized treatment choice may potentially confound patient outcome, contributing to selection bias in the present study. Finally, due to the lack of insurance support in Taiwan, less than 1% of our patients received FOLFIRINOX or gemcitabine plus nab‐paclitaxel, which are the standard first‐line chemotherapy regimens for pancreatic cancer in Western countries. Thus, the applicability of the GS model as a prognostic model in Western patients with APC remains unknown.

## 5 CONCLUSION

The results of our study validated the GS model's competence in predicting survival outcome in a large cohort of patients with unresectable or metastatic PC. Moreover, the results supported the clinical application of the GS model in the first‐line treatment of APC with gemcitabine‐based regimens. In addition, our results suggested that the GS model may be used to predict tumor response and provide guidance when choosing between monotherapy or combination chemotherapy among patients in different prognostic groups in the GS model.

## CONFLICT OF INTEREST

The authors declare that no competing interests exist.

## Data Availability

The data that support the findings of this study are available from the corresponding author upon reasonable request.
