# Supplementary figures and images for: Validation and application of a prognostic model for patients with advanced pancreatic cancer receiving palliative chemotherapy
Source: Cancer Med. 2019 Aug 6;8(12):5554–63. doi: 10.1002/cam4.2483 (PMC6745849; doi:10.1002/cam4.2483)

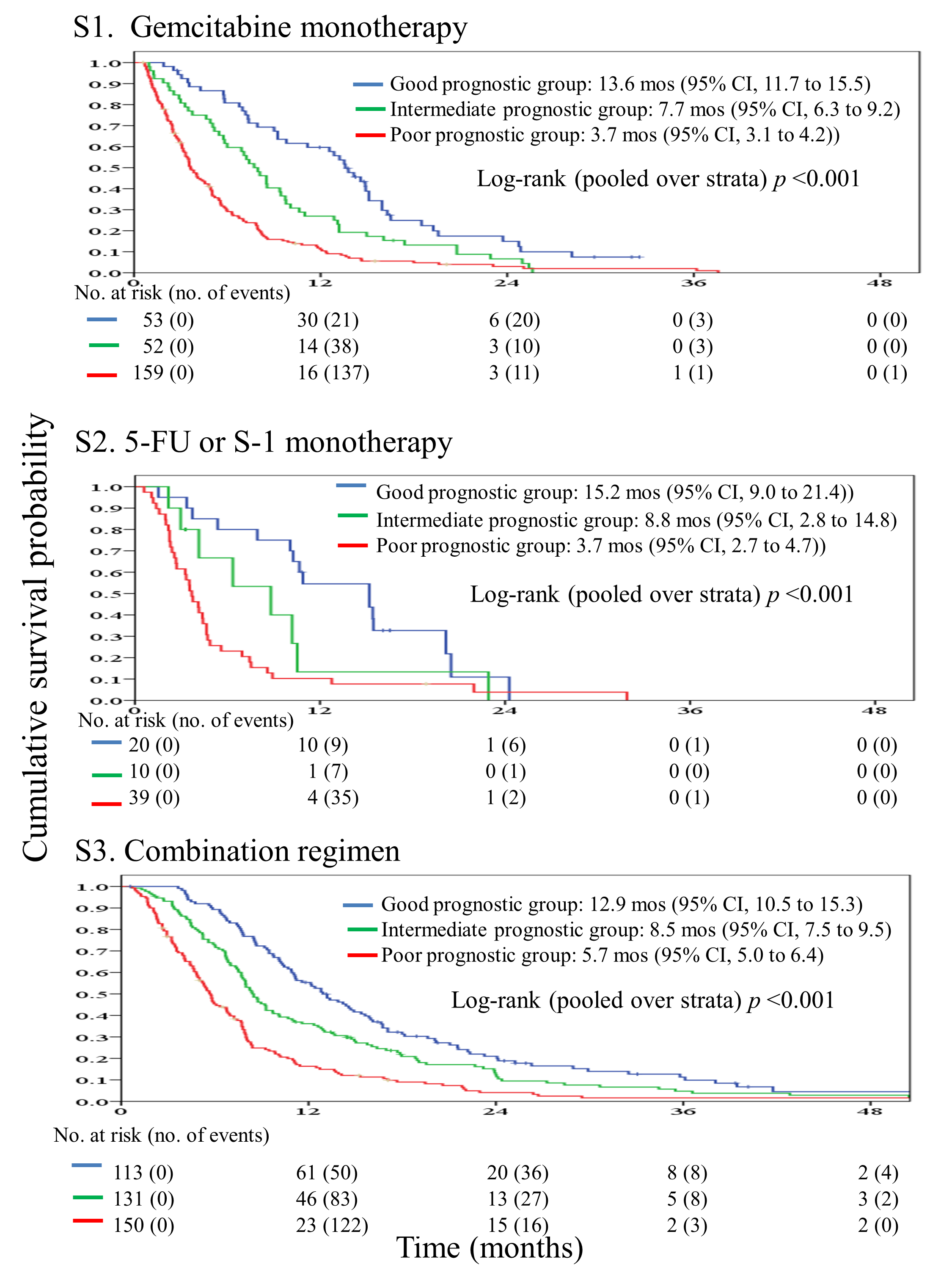

Supplement: Supplementary file 1 [file CAM4-8-5554-s001.tif]
